# Selective Conditions for a Multidrug Resistance Plasmid Depend on the Sociality of Antibiotic Resistance

**DOI:** 10.1128/AAC.02441-15

**Published:** 2016-03-25

**Authors:** Michael J. Bottery, A. Jamie Wood, Michael A. Brockhurst

**Affiliations:** a Department of Biology, University of York, York, United Kingdom; b Department of Mathematics, University of York, York, United Kingdom

## Abstract

Multidrug resistance (MDR) plasmids frequently carry antibiotic resistance genes conferring qualitatively different mechanisms of resistance. We show here that the antibiotic concentrations selecting for the RK2 plasmid in Escherichia coli depend upon the sociality of the drug resistance: the selection for selfish drug resistance (efflux pump) occurred at very low drug concentrations, just 1.3% of the MIC of the plasmid-free antibiotic-sensitive strain, whereas selection for cooperative drug resistance (modifying enzyme) occurred at drug concentrations exceeding the MIC of the plasmid-free strain.

## TEXT

Antibiotics are critical to modern medicine, but their widespread use and misuse have led to the evolution of strains resistant to most commonly used antibiotics (1, 2). Antibiotic resistance has become a major threat to global health, with multidrug-resistant (MDR) bacteria observed globally (3). Environmental antibiotic resistance genes (ARGs) are a major source of clinical resistance (4). ARGs can be selected for at very low concentrations of antibiotics, far below the MIC of sensitive cells (5, 6), with antibiotic contamination at sub-MICs being proposed as the main driving force behind environmental selection for resistance (7–9). However, ARGs can encode qualitatively different forms of resistance, ranging from selfish to cooperative. Selfish drug resistances confer a benefit only to the individual cell harboring them, for example, efflux pumps, reduced membrane permeability, and antibiotic target alteration (10, 11). In contrast, cooperative antibiotic resistance benefits both the resistant cell and surrounding cells, whether they are resistant or not. For example, modifying enzymes, such as β-lactamases, inactivate the antibiotic through hydrolysis, decreasing its environmental concentration. Localization of the β-lactamase enzyme in the periplasmic space may enhance the share of the benefit for the resistant cell, but nevertheless, the decrease in the overall environmental concentration of antibiotic will benefit both resistant and sensitive cells (12). We hypothesized that the sociality of drug resistance alters the selective conditions for the spread of ARGs (13, 14). Specifically, because the benefits of selfish drug resistance are directed solely to the resistant cell, whereas the benefits of cooperative drug resistance are shared between resistant and sensitive cells, we predicted that selfish drug resistance should be selected at lower relative drug concentrations (i.e., % of the sensitive MIC) than those for cooperative resistance.

Multiple ARGs are frequently clustered together onto conjugative plasmids, including combinations of selfish and cooperative drug resistance (15). How combinatorial antibiotic usage selects for MDR plasmids is not clear, especially for combinations of antibiotics requiring qualitatively different modes of drug resistance, such as selfish or cooperative drug resistance. Here, we tested how the sociality of drug resistance and single versus combined antibiotic treatment altered the selective conditions for the MDR plasmid RK2 (16) in Escherichia coli MG1655. RK2 carries genes encoding both cooperative ampicillin resistance, mediated by a β-lactamase, and selfish tetracycline resistance, mediated by an efflux pump. We report that the selfish drug resistance is selected for at far lower relative antibiotic concentrations than those for cooperative drug resistance and that combined antibiotic selection is additive, showing no interaction.

**FIG 1:**
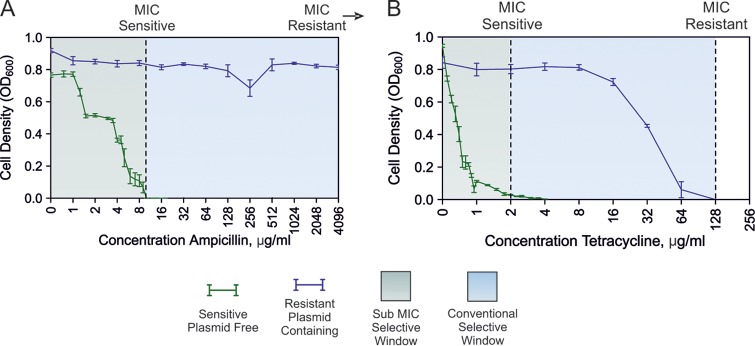
Cell density (optical density at 600 nm [OD_600_]) of sensitive plasmid-free bacteria (green line) and resistant plasmid containing bacteria (blue line) as a function of ampicillin concentration (A) and tetracycline concentration (B) after 24 h of growth in monoculture. The error bars show standard error of the mean (SEM) values (*n* = 6). The area shaded in green shows the sub-MIC selective window, and the area shaded in blue shows the selective window conventionally thought to select for resistance.

**FIG 2:**
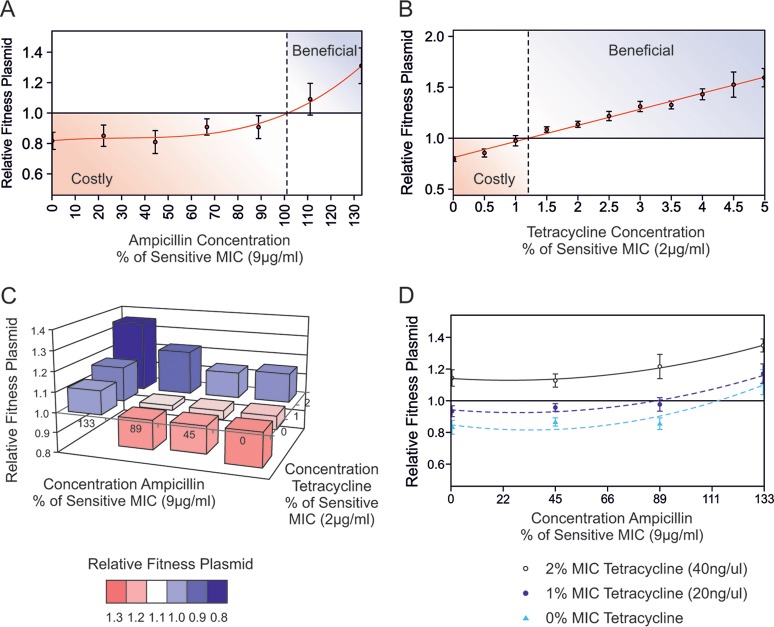
Fitness reaction norms as a function of antibiotic concentration during competition experiments between E. coli harboring the RK2 plasmid and isogenic plasmid-free sensitive strains. Competition in the presence of ampicillin (A) and tetracycline (B) is shown, and the red lines show a fitted regression. Dashed lines represent antibiotic concentrations predicted to select for RK2 plasmid. (C and D) Fitness reaction norms of combination treatments with both ampicillin and tetracycline during competition experiments between E. coli harboring the RK2 plasmid and plasmid-free strains; these are alternative visualizations of the same experimental data. There is no significant interaction of antibiotic treatments upon the relative fitness (*F*_1,68_ = 0.2395, *P* = 0.6261), indicating that treatments were noninteracting and additive. The error bars in panels A, C, and D show the SEM values (*n* = 6). Antibiotic concentrations are shown as percentages of the MIC for sensitivity.

Conventionally, ARGs are thought to be positively selected at antibiotic concentrations exceeding the MIC of sensitive cells in monoculture (17) (i.e., the conventional selective window, Fig. 1). To determine whether the sociality of resistance affected the selection window for the RK2 MDR plasmid, we estimated the relative fitness of plasmid-bearing versus isogenic plasmid-free cells by direct competition, according to standard methodology (see the supplemental material). In the absence of antibiotics, the plasmid imposed a significant cost of carriage, decreasing the fitness of E. coli by 19% (Fig. 1A and B, *t* test, *P* < 0.001, *t* = −9.8674, *df* = 23). An intrinsic cost is often associated with plasmid carriage when accessory traits are not under positive selection due to cellular disruption and increased transcriptional load (18). Cooperative ampicillin resistance was positively selected at ampicillin concentrations exceeding the MIC of plasmid-free sensitive E. coli strains (Fig. 2A). Importantly, sensitive cells were able to maintain positive growth in mixed cultures at ampicillin concentrations that completely inhibited their growth in monoculture (>8 μg/ml; Fig. 1A; see also Fig. S4 in the supplemental material), justifying the assignment of ampicillin resistance as cooperative. Thus, cooperative resistance permits the persistence of a sensitive subpopulation beyond the sensitive MIC due to the inactivation of the antibiotic, potentially allowing reinvasion by sensitive cells once the antibiotic concentration is sufficiently reduced by the action of resistant cells.

In contrast, selfish tetracycline resistance was positively selected at tetracycline concentrations of just 1.3% of the MIC of sensitive E. coli (Fig. 2B). Indeed, at concentrations of tetracycline that were >10% of the MIC of sensitive E. coli, the resistant plasmid bearers competitively excluded the plasmid-free bacteria, with no plasmid-free cells observable (see Fig. S1 in the supplemental material). This is despite the fact that plasmid-free E. coli survived at these tetracycline concentrations when grown alone (Fig. 1B). Our data suggest that selfish tetracycline resistance is positively selected in the sub-MIC selective window at very low tetracycline concentrations, similar to those observed in the natural environment (19).

When ampicillin and tetracycline were applied in combination, there was no significant interaction (*F*_1,68_ = 0.2395, *P* = 0.6261), indicating that when these two antibiotics were used in combination, their selective effects were independent and additive (Fig. 2C). This means that very low concentrations of tetracycline were sufficient to completely mask the population-level effects of cooperative ampicillin resistance. With increasing tetracycline concentrations, the ampicillin concentration positively selecting for the MDR plasmid shifted to lower and lower sub-MIC levels, reducing the window of selective conditions under which sensitive cells could persist (Fig. 2D).

Residues of multiple antibiotics are commonly found to contaminate the same environments at low concentrations (19, 20). These combinations, and particularly the presence in the environment of antibiotics, like tetracycline, targeted by selfish efflux-mediated resistance will select for the spread of MDR plasmids and competitive exclusion of sensitive cells. This is despite being present at concentrations far below the level required to positively select resistance individually. This adds further evidence that ARGs, whether chromosomal or carried by plasmids, can be positively selected at antibiotic concentrations far below the MIC of sensitive strains (5, 6, 9).

Our study has a number of possible limitations: First, it is possible that other factors in addition to sociality may have contributed to differences in the fitness reaction norms of the antibiotics, including the contrasting effects of sub-MICs on monoculture densities, and the fact that ampicillin is bactericidal while tetracycline is bacteriostatic. Second, we use exemplars of cooperative and selfish resistance, but more research will be required to test the importance of sociality on the selective conditions for other resistance mechanisms.

Here, we show that the extent to which an ARG is positively selected at sub-MICs depends upon the sociality of the mechanism of drug resistance. Cooperative ampicillin resistance is positively selected at ampicillin concentrations exceeding the MIC, whereas selfish tetracycline resistance is positively selected at 100-fold-lower relative drug concentrations. This striking difference in the selective window for ARGs located on the same MDR plasmid probably arises because of the population-level effects of the ARGs: cooperative ampicillin resistance allowed sensitive bacteria to survive at concentrations above their MIC by reducing the ampicillin concentration and sharing the benefits of resistance, whereas selfish tetracycline resistance drove the complete competitive exclusion of sensitive cells at >10% of the MIC due to the exclusively individual benefits of efflux-mediated resistance. Combining the two antibiotics at concentrations that would not normally select for resistance individually selects for both resistances and the spread of the MDR plasmid. Taken together, these findings suggest that selfish efflux-mediated drug resistances are likely to be especially important for the selective maintenance and spread of MDR plasmids.

## Supplementary Material

Supplemental material
