# Supplementary material for: Selective Conditions for a Multidrug Resistance Plasmid Depend on the Sociality of Antibiotic Resistance
Source: Antimicrob Agents Chemother. 2016 Mar 25;60(4):2524–7. doi: 10.1128/AAC.02441-15 (PMC4808222; doi:10.1128/AAC.02441-15)
Supplement: Supplemental material [file supp_60_4_2524__index.html]

Selective Conditions for a Multidrug Resistance Plasmid Depend on the Sociality of Antibiotic Resistance — Supplemental material 

# Selective Conditions for a Multidrug Resistance Plasmid Depend on the Sociality of Antibiotic Resistance

## Supplemental material

- Supplemental file 1 -

  Supplemental Methods and Fig. S1-S4

  PDF, 209K
