# Supplementary material for: Selective Conditions for a Multidrug Resistance Plasmid Depend on the Sociality of Antibiotic Resistance
Source: Antimicrob Agents Chemother. 2016 Mar 25;60(4):2524–7. doi: 10.1128/AAC.02441-15 (PMC4808222; doi:10.1128/AAC.02441-15)
Supplement: Supplemental material [file AAC.02441-15_zac004165001so1.pdf]

SUPPORTING INFORMATION FOR

SELECTIVE CONDITIONS FOR A MULTIDRUG RESISTANCE PLASMID DEPEND ON THE SOCIALITY OF ANTIBIOTIC RESISTANCE

SUPPLEMENTARY METHODS

(a) Strains, culture conditions and MIC measurements

Isogenic *E. coli* MG1655 chromosomally labelled with GFP or mCherry, at the *attB* lambda attachment site through λ red homologous recombination, were provided by the Van Der Woulde lab (University of York). The RK2 was introduced to the marker strains through conjugation from *E. coli* MV10 provided by the Thomas lab (University of Birmingham). All cultures were conducted in Oxoid® Nutrient Broth (NB) at 37°C, shaken at 180 rpm. To measure MICs, cultures were grown over night until stationary phase in 5ml Nutrient Broth, the saturated cultures were then diluted into 96 well plates to a initial density of $5{\times}10^5$ CFU/ml. Cultures were grown for 24 hours with OD_{600} measured at the end point. The MIC was defined at the lowest concentration, which inhibited all bacterial growth. These conditions informed the antibiotic conditions used to in competitive fitness assays.

(b) Competitive fitness assays

The relative fitness of plasmid bearing versus plasmid free cells was estimated by direct competition experiments. To control for fitness effects of the fluorescent markers, competitions were performed with the plasmid carried in the MG1655-gfp background against plasmid-free MG1655-mcherry for half of the replicates, and vice versa for the other half of the replicates. Competitions were conducted across antibiotic concentration gradients, ranging from no antibiotic to 12µg/ml ampicillin or 250ng/ml tetracycline. Six replicates of each antibiotic concentration were conducted. The competitions were initiated with 1:1 mixtures of plasmid-bearing against plasmid-free strains from overnight cultures at an initial density of approximately $5{\times}10^5$ CFU/ml. To gain exact viable cell counts, dilutions of culture were spread on to nutrient agar plates. Competition cultures were grown for 24 hours, and final densities calculated via plate counts, strains were distinguished through detection of fluorescent markers using Zeis Stereo Lumar v12 microscope. The relative fitness of plasmid-bearing bacteria was calculated as a ratio of Malthusian parameters: $W_{\mathrm{plasmid}}=\ln(N_{\mathrm{final,plasmid}}/N_{\mathrm{initial,plasmid}})/\ln(N_{\mathrm{final,free}}/N_{\mathrm{initial,free}})$ [1] .No significant difference between growth dynamics of marker strains was observed (Fig. S2 supplementary material) along with no significant difference in relative fitness (Fig. S3).

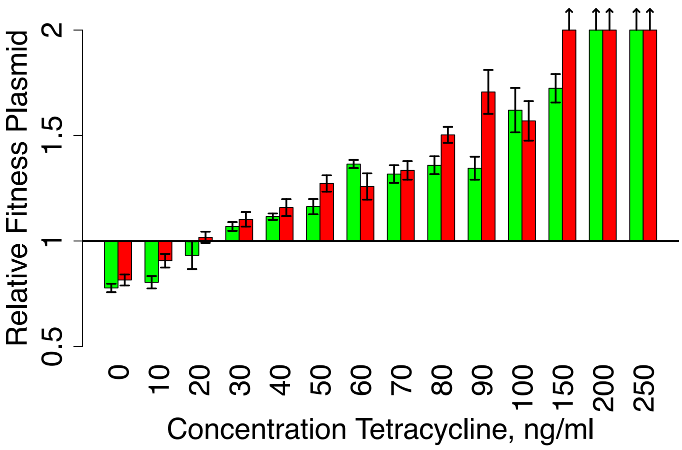

FIG S1
High concentrations of tetracycline cause extinction of sensitive population when in competition. Green bars are the RK2 plasmid in MG1655::gfp background competed against MG1655::mcherry and red bars are the RK2 plasmid in MG1655::mcherry background competed against MG1655::gfp. Upward arrows represent a relative fitness above 2 i.e. no observable sensitive bacteria within the population. Error bars represent SEM (n=3).

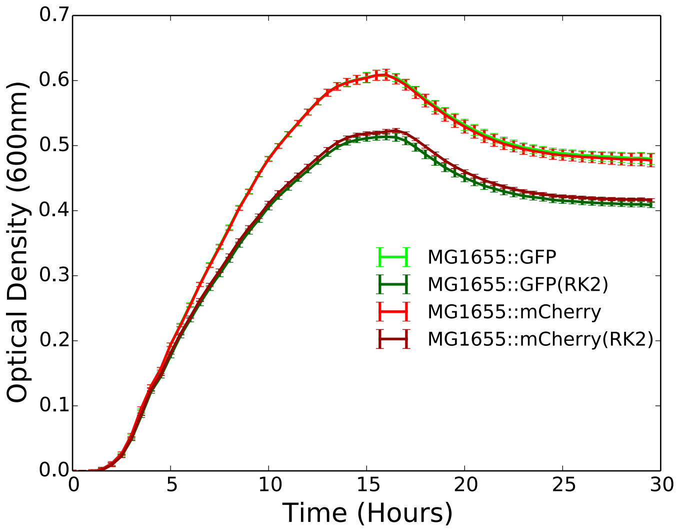

FIG S2
Growth curves of marked strains show no difference in growth dynamics between marked strains, with or without RK2 plasmid. Bacteria were grown in 96-well plates at 37°C with periodic shaking in a Tecan Infinite 200 Pro plate reader, with OD_{600} measured every 30 mins. Error bars represent SEM (n=6).

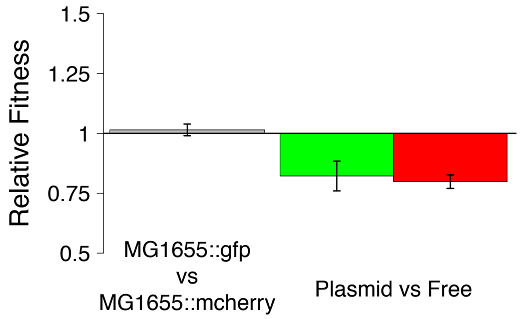

FIG S3
No significant difference in fitness of MG1655::gfp strain and MG1655::mcherry strain (t test t = 0.6001, df = 11, p-value = 0.5606) or between MG1655::gfp(RK2) and MG1655::mcherry(RK2) (two sample t test t = 0.5186, df = 16, p-value = 0.6944) when competed in antibiotic free media. Green bar represents RK2 plasmid in MG1655::gfp background competed against MG1655::mcherry and red bars are RK2 plasmid in MG1655::mcherry background competed against MG1655::gfp. Error bars represent SEM (n=6).

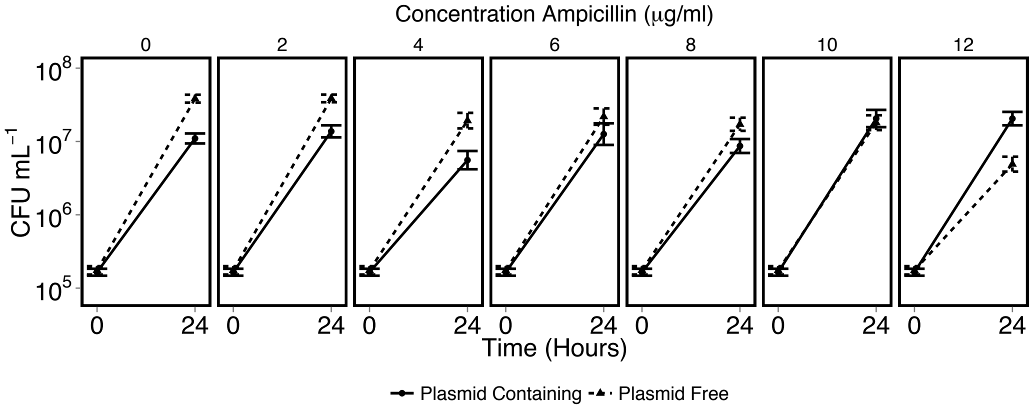

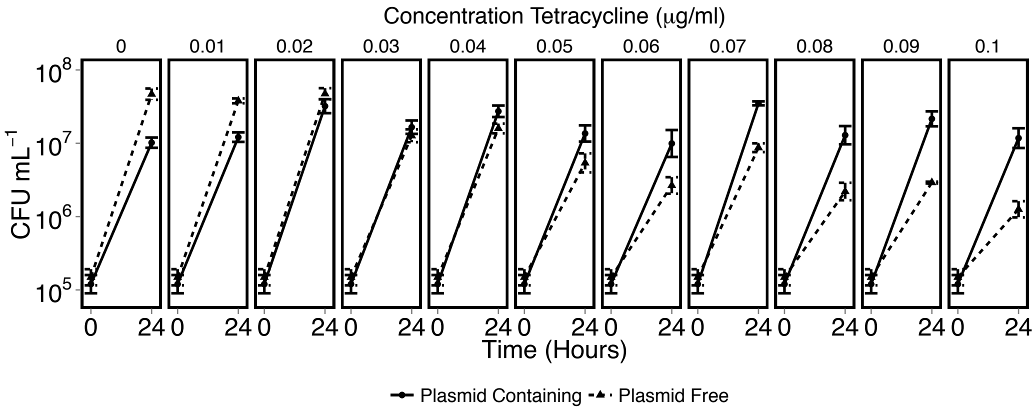

FIG S4
Plots of cell densities of plasmid free and RK2 plasmid containing populations in competition, these data were used to calculate the relative fitness of plasmid bearing strain (figure 2 in text). Note that relative fitness (*W*) relies on positive growth of both competing strains. Error bars represent SEM (n=6).

REFERENCES

1. **Lenski RE**, **Rose MR**, **Simpson SC**, **Tadler SC**. 1991. Long-Term Experimental Evolution in Escherichia coli. I. Adaptation and Divergence During 2,000 Generations. Am Nat **138**:1315–1341.
